# Experimental Outcrossing in *Agaricus bisporus* Revealed a Major and Unexpected Involvement of Airborne Mycelium Fragments

**DOI:** 10.3390/jof8121278

**Published:** 2022-12-06

**Authors:** Banafsheh Jalalzadeh, Gérard Barroso, Jean-Michel Savoie, Philippe Callac

**Affiliations:** 1 INRAE, Mycology and Food Safety (MycSA), F-33882 Villenave d’Ornon, France; 2 Department of Ecology of Agricultural Plants, Arid Environments Research Centre, Islamic Azad University, Mashhad 9187147578, Iran

**Keywords:** Buller phenomenon, CAPS marker, haplotype marker, heteromorphism, hybrid, propagule

## Abstract

*Agaricus bisporus* var. *bisporus*, the button mushroom, has a predominantly pseudohomothallic life cycle. Most of its spores are heterokaryotic and give rise to fertile heterokaryons. However, previous studies have suggested that outcrossing should not be rare in wild populations. In order to discover how outcrossing occurs, we experimentally favored it between aerial propagules of a fruiting donor mycelium and a delayed receiver mycelium that only invaded culture trays. Two donor/receiver pairs were studied, and potentially hybrid basidiomata collected on the receiver trays were analyzed with a mitochondrial marker, two unlinked nuclear CAPS markers, then haplotype markers based on DNA sequences obtained after PCR cloning of the rDNA ITS region and the *fruk* gene. For one of the two pairs, most basidiomata were hybrids between the donor and the receiver. Genotyping of the hybrids revealed only two genotypes consistent with outcrossing involving airborne mycelium fragments rather than basidiospores. The resident receiver heterokaryon that provided its mitochondria to the hybrid basidiomata is suspected to have had a trophic contribution to their growth and successful fruiting. The high level of heterozygosity and the cultivar introgression previously revealed in wild populations of this pseudohomothallic species may result from outcrossing involving airborne pieces of mycelium.

## 1. Introduction

The button mushroom, *Agaricus bisporus* (J.E.Lange) Imbach, is a secondary decomposer [1] which naturally grows in forests, mostly in cypress or spruce litter, in sandy semi-arid habitats such as in deserts or dunes, and in more precarious habitats on organic wastes such as horse manure or grass clippings [2]. In *A. bisporus*, three varieties with different types of life cycles have been proposed, as summarized by Savoie et al. [3]. This review has already addressed the simple and fundamental question of whether, when, and how outcrossing occurs in natural populations of *A. bisporus*. This question mainly concerns *A. bisporus* var. *bisporus* and not the other two varieties. Indeed, in *A. bisporus* var. *eurotetrasporus*, Callac and Guinb., outcrossing does not occur since this rare variety is homothallic [4]. In contrast, in the variety *A. bisporus* var. *burnettii* Kerrigan and Callac, which is known only from the Sonoran Desert of California, USA, outcrossing is expected to frequently occur because its life cycle is amphithallic (i.e., both heterothallic and pseudohomothallic) but predominantly heterothallic [5]. In this variety, most basidia are tetrasporic and, correlatively, most basidiospores are haploid, giving rise to homokaryons. Therefore, it is expected that plasmogamy could easily occur between sexually compatible homokaryons to restore a fertile (capable of fruiting) heterokaryotic mycelium. In *A. bisporus*, the system of sexual incompatibility is unifactorial with at least 14 alleles at a centromere-linked locus *MAT* [6,7,8]. The present study focuses on the variety *A. bisporus* var. *bisporus* to which most of wild populations and cultivars belong. This variety also has an amphithallic life cycle; however, contrarily to var. burnetii, it is predominantly pseudohomothallic (=secondarily homothallic, [9]). In this variety, most basidia are bisporic and, correlatively, most basidiospores give rise to fertile heterokaryons. Indeed, in bisporic basidia of *A. bisporus*, non-sister post-meiotic nuclei preferentially migrate in the same spore, and since the *MAT* locus is centromere-linked they bear the two different parental mating type alleles. In addition, in the heterokaryotic spores of *A. bisporus* var. *bisporus*, most of the parental heterozygosity is maintained due to recombination being restricted at chromosome ends [10]. Since few basidia are tetrasporic, only a small fraction of spores is homokaryotic, and thus crosses between homokaryons seem unlikely. However, for at least two reasons, outcrossing necessarily occurs: first, to explain the introgression of cultivars in wild populations [11]; second, to explain the high level of heterozygosity reported in certain populations of *A. bisporus* var. *bisporus* [11,12].

Population genetic analyses based on harvested basidiomata were unable to infer when and how outcrossing occurred for two main reasons. The first reason is that occurrence of *A. bisporus* basidiomata in the wild is unpredictable and occasional [13]. Although in some collecting sites, dense and variable populations have been sampled for two consecutive years [12], it is likely that many mycelia could not be isolated because they did not fruit simultaneously at the time of collection. In the latter study, there was no correlation between genetic distance and physical distance and little evidence of vegetative clonality (maximum distance of 1.3 m). In addition, even among basidiomata of several clusters collected at less than 10 cm from each other, different nuclear and/or mitochondrial genotypes were observed. This was why we decided to experimentally simulate outcrossing conditions with putative parents growing relatively close together.

The second reason for the failure of population genetic analyses to understand how outcrossing occurs is the diversity and the complexity of the outcrossing processes. Outcrossing occurs between two mycelia; however, direct plasmogamy between mycelium and spore is not excluded. The encounter is most likely to occur between a mycelium installed on the soil as a resident mycelium, and other mycelia growing from airborne spores or mycelium fragments. Regardless of the homokaryotic or heterokaryotic status of the parental mycelia, if the resulting mycelium after plasmogamy has a nucleus of each of these mycelia, the cross is successful and the new heterokaryon is often called a “hybrid”. In addition, hybrid hyphae produce a new mycelium because in *A. bisporus*, unlike many species of Basidiomycota, nuclear migration in the parental mycelia generally does not occur.

In wild populations of *A. bisporus* var. *bisporus*, evidence for pseudohomothallic reproduction appears to depend on the studied populations. On the one hand, Kerrigan [14] found some evidence for the existence of pseudoclonal lineages resulting from successive pseudohomothallic generations, in populations of North America. In such lineages, the initial heterozygosity remains highly conserved. On the other hand, Xu et al. [12] found, contrary to expectation, limited evidence for pseudohomothallic reproduction or inbreeding but abundant evidence for outcrossing and recombination in two local French populations studied over a period of two years. Two explanations were proposed by the authors: first, despite the small fraction of homokaryotic spores, they could play an important role knowing that a basidioma produces about one billion spores. In addition, counting the percentages of n-spored basidia (n varying from 1 to 4) in cultivated fruiting bodies of 215 wild isolates from France and hypothesizing that trisporic basidia would produce two homokaryotic spores and one heterokaryotic spore, Callac et al. [15] estimated the fraction of homokaryotic spores to be on average 19%. However, due to lethal or deleterious alleles, the fraction of mycelia derived from homokaryotic spores is probably much lower. The second explanation was that mating between homokaryon and heterokaryon, also called the Buller phenomenon [16], or between heterokaryons could contribute to the generation of novel genotypes. Xu et al. [17] demonstrated that such pairing can produce new hybrid heterokaryons, but also that somatic recombination including crossing over can occur during these processes in Petri dishes. In subsequent studies, Callac et al. [18,19,20] repeatedly found hybrid basidiomata on compost trays that were simultaneously inoculated with a homokaryon (as grain spawn) from one parent and spores from the spore print of another parent. Genotype analyses showed that all basidiomata resulted from crosses between the inoculated homokaryotic mycelium and heterokaryotic spores (or mycelia that derived from them), via the Buller phenomenon. Interestingly, in these experiments, pseudohomothalism completely failed in the co-inoculated culture trays as well as in the control trays only inoculated with spores. The authors concluded that in those conditions the spores apparently needed a mycelium to stimulate their germination. In other terms, these experiments showed that when encountered a homokaryotic mycelium, the fertile heterokaryotic spores preferentially crossed via the Buller phenomenon (homo × hetero) and thus contributed to the fruiting of hybrid basidiomata, rather than growing and fruiting on their own. However, in the wild, it is unlikely to find a widely extended homokaryotic mycelium of *A. bisporus* var. *bisporus* in the ground or this should be ephemeral and is more likely to encounter a heterokaryotic mycelium. Knowing if this latter scenario may occur significantly in natural populations may improve our understanding of their biodiversity.

In order to investigate the reproductive behavior of *A. bisporus* in the wild, we decided to set up an experiment under semi-controlled environment. Specifically, we investigated the occurrence of outcrossing between a preinstalled ‘receiving’ heterokaryotic mycelium (the predominant mycelial stage of Basidiomycota in the wild) and airborne propagules from a fruiting ‘donor’ heterokaryon at sporulating stage. Our immediate and longer-term goal is to understand how outcrossing is involved in the genetic diversity, high heterozygosity and cultivar introgression observed among wild-collected basidiomata of *A. bisporus*.

We used two genetically unlinked nuclear DNA markers and one mitochondrial marker. Surprisingly, the hybrid basidiomata collected on the culture trays of a receiver strain resulted from crosses between this strain and airborne pieces of mycelium from the donor strain. It was unexpected to find that hybrid basidiomata resulted mainly from crosses between the two heterokaryons and that spores were not or little involved.

## 2. Materials and Methods

### 2.1. Strains and Grain Spawn Preparation

The three strains of *A. bisporus* var. *bisporus* Bs177, Bs243 and Bs256, used in this study, are subcultures of mycelia isolated by tissue culture from basidiomata collected in the wild under *Hesperocyparis macrocarpa* (Hartw.) Bartel (syn. *Cupressus macrocarpa* Hartw.). They belong to two French populations of *A. bisporus* located in cypress woods near Lorient (Bs177 and Bs243) and Saint-Malo/Dinard (Bs256), which were previously studied by Xu et al. [11,21]. In *A. bisporus* var. *bisporus* [9], as in most Basidiomycota, such isolates are heterokaryotic [n + n], and capable of fruiting. Indeed, they possess the same two constituent haploid nuclei as the basidioma from which they were isolated, and consequently, two different alleles at the mating type locus. Strains were kept under liquid nitrogen in the “Collection du Germoplasme des Agarics à Bordeaux” (CGAB) until 2021. This collection no longer exists but about 400 strains from it have been transferred and are now available at CIRM-CF in Marseille, France (https://www.cirm-fungi.fr/, accessed on 6 November 2022). The strains Bs177, Bs243 and Bs256 are in the catalog of this Biological Resource Center (BRC) with accession numbers 2712, 3171 and 2827, respectively. They had been selected among more than 600 strains of our collection because of their regional origin, their optimal mycelial growth rate, their genetic variability for the used markers, and owing to their different cap colors we expected to detect the putative resulting hybrids more easily.

To prepare grain spawn, each strain was first cultured in Petri dishes (90 mm diameter) containing a solid complete yeast medium [9] supplemented with 0.1% of compost extract during 14 days at 25 °C. For each strain, grain spawn were prepared in plastic boxes by inoculating rye grains (160 g of grains previously autoclaved at 121 °C for 20 min) with mycelial colonies from two previously invaded Petri dishes, followed by incubation for 25 days at 25 °C.

### 2.2. Experimental Outcrossing

In order to perform outcrossing between strains, two types of culture trays were started 20 days apart. The first types of culture trays were prepared to be invaded and to produce mature basidiomata. This donor mycelium and its basidiomata are intended to generate a cloud of airborne propagules consisting of both basidiospores and mycelium fragments. The second types of culture trays were designed to be first invaded by the propagule-receiving mycelium and then exposed to the cloud of airborne propagules from the donor. The basidiomata produced on these trays will be examined phenotypically and genotypically to determine whether they were produced by the receiver mycelium, by the propagules from the donor mycelium or by crosses between the receiver and the donor.

For the donor strain Bs243, culture trays were performed by inoculating six trays (48 × 22 × 20 cm) containing 8 kg of compost (commercial production, Renaud & Fils SARL Company, Avy, France) with 80 g of grain spawn. For each of the two receiver strains (Bs177 and Bs256), two larger trays (60 × 40 × 14 cm) containing 10 kg of compost were inoculated with 100 g of grain spawn. Experiments were also performed with either Bs117 or Bs256 as donors; however, these are not reported because no hybrid was detected.

**Figure 1:**
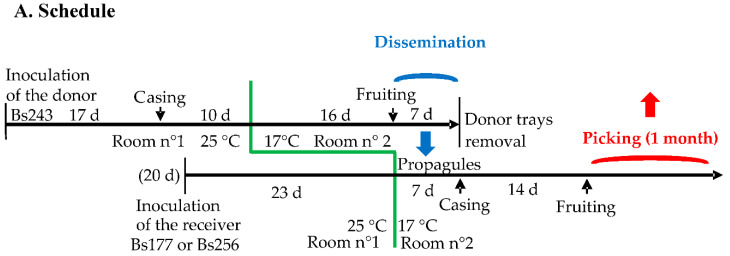

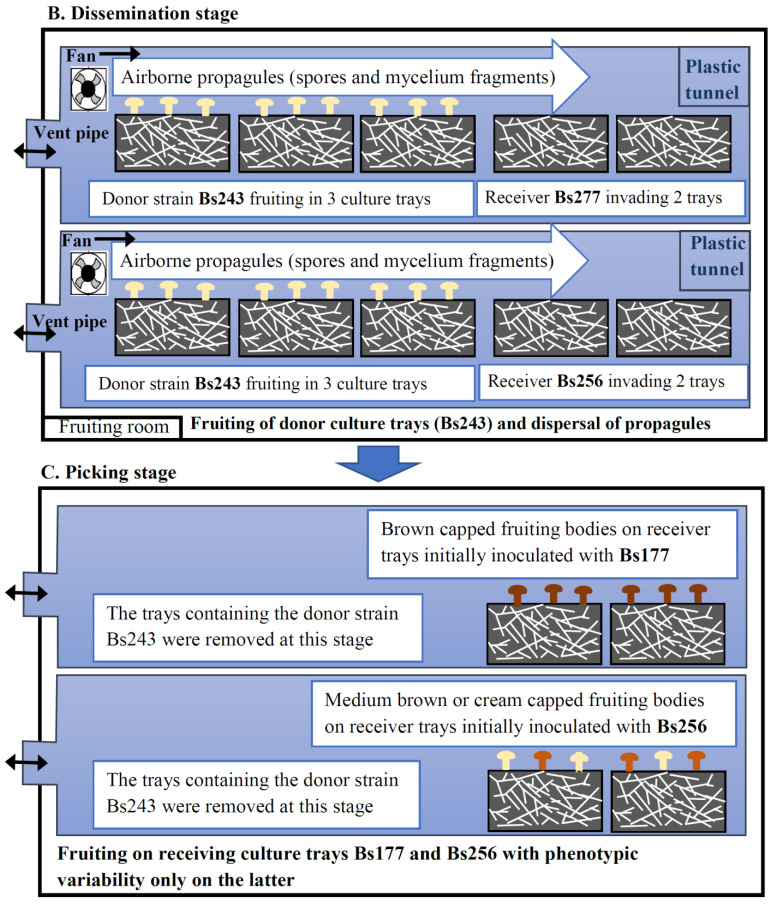
Experiments favoring outcrossing between heterokaryons growing close to each other. (**A**). Schedule of the staggered cultures of donor and receiving mycelia in the incubation room (n°1) and the fruiting room (n°2). The blue arrow shows the 7-day period during which propagules of the donor are disseminated to the receiver (dissemination stage). The red arrow shows the picking period on receiver trays (picking stage). (**B**). Dissemination stage in the fruiting room in which two couples of donor/receiver heterokaryons were isolated in two plastic tunnels. The donor strain (Bs243) was fruiting in first and its airborne propagules were spread on the receiver strain (Bs177 or Bs256) in each of the two tunnels, respectively. At this step, the receiver trays were invaded by these strains but were not yet fruiting because they had been inoculated 20 days after the donor trays. This stage favors outcrossing between propagules from the donor and the receiver heterokaryotic mycelium. (**C**). Picking stage on receiver trays. On Bs177, all basidiomata were dark brown in color with the phenotype and the genotype of Bs177; in contrast, on trays initially inoculated with Bs256, cap color of the basidiomata was variable, suggesting that some of them could result from hybridization between Bs243 and Bs256, which has been confirmed with genetic markers.

The process of staggered cultures of donor and receiving trays is showed in Figure 1A. After inoculation of the grain spawn with a 20-day lag for the receiver, the culture trays were placed in the incubation room n°1 at 25 °C. When the compost was invaded, the trays were moved to the controlled environment room n°2 for fruiting at 17 °C. A casing layer was added on compost when the latter was invaded by the mycelium. This layer was a mixture of sand, peat and limestone in equal volumes, previously autoclaved at 121 °C for one hour. As indicated in Figure 1A, casing was delayed for the receiver trays because, for one week in room n°2, their mycelium had to be visible on the surface and thus potentially able to receive propagules from the donor trays that were producing mature basidiomata at this time. Within fruiting room n°2, three donor trays of Bs243 and two receiving trays of Bs177 or Bs256 were lined up and enclosed in two plastic tunnels, respectively, as indicated in Figure 1B. These tunnels were, in fact, two long, large transparent plastic tubes that could be opened only at their ends.

For one week, a small fan was installed in the plastic tunnel (Figure 1B) to simulate wind dispersing propagules from the donor trays to the receiver trays. In order to check for the occurrence of effective contacts between receiver mycelia and disseminated propagules (basidiospores or mycelium fragments) from the donor strain, a trapping adhesive strip was installed along the surface of each receiving tray. The presence of the propagules on the adhesive strips was then verified by light microscopy. In addition, for the same week, intermittent aeration of the cultures was carried out by unrolling and opening an unused segment of each plastic tunnel outside the fruiting room to avoid contamination between the receiving strains of both tunnels. After one week, donor culture trays were removed from the tunnels and the casing layer was added onto the receiving trays (Figure 1C). About 14 days after the addition of the casing layer, fruiting bodies grew on the receiving trays. To avoid spreading their basidiospores, fruiting bodies were harvested before cap opening. The preparation of the grain spawn and the simulation experiment required at least four and a half months.

### 2.3. DNA Extraction, PCR Amplification and Sequencing

Extraction of genomic DNA from 0.2 g of a lyophilized basidioma was performed by the “miniprep” CTAB method [22]. PCR reactions were performed using GoTaq^®^ DNA polymerase, according to the supplier’s recommendation (Promega, Madison, WI, USA) with pairs of primers. For the internal transcriber spacer (ITS) region of nuc rDNA (ITS1 + 5.8S + ITS2), the forward and reverse (5′-3′) primers were as follows: ITS5: (GGAAGTAAAAGTCGTAACAAGG and ITS4: TCCTCCGCTTATTGATATGC [23]. For the 5′ region of the fructosamine-kinase gene (*fruk*) the primers were PR6F: AGGTGACATGTCAGAAGCGC and PR6R: CAATCTCAAGCTTGCCTGG. This marker was named PR6 in Imbernon et al. [24] and *frk* in Jalalzadeh et al. [25]. Amplification conditions were as follows: 95 °C for 5 min, 35 cycles × [95 °C for 30 s, 54 °C (ITS5/ITS4) or 58 °C (PR6U/PR6R) for 30 s, 72 °C for 1 min], 72 °C for 5 min. PCR amplification of the mitochondrial *iAbi11* sequence was achieved using i11U: GGTTCTTGGTCAAATTAAA and i11R: CTATACCAAATCCTGGTAT primers [25] as follows: 95 °C for 5 min, 35 cycles × [95 °C for 30 s, 50 °C for 30 s, 72 °C for 3 min], 72 °C for 5 min. When required, PCR products were sequenced by Cogenics-Genome Express (Grenoble-France). The produced sequences have been deposited at the NCBI database. The relevant names and accession numbers of the ITS and *fruk* sequences of the parental strains are indicated at Table 1 and Table 2, respectively. (For accession numbers, see also the Data Availability Statement.)

### 2.4. Nuclear Cleaved Amplified Polymorphic Sequence (CAPS) and Nuclear Haplotype Markers

These markers were used to establish the genotypes of the parent strains and harvested basidiomata. They are based on nuclear single nucleotide polymorphisms (SNPs) that were identified in multiple sequence alignments of the ITS region or the *fruk* gene using CLUSTALW [26]. To design CAPS markers, nuclear sequences were analyzed with the help of a restriction map generator (https://www.algosome.com/resources/restriction-map/enzyme-digest.php, accessed on 6 November 2022). PCR products of the ITS region or *fruk* gene were analyzed by 1% agarose gel electrophoresis after digestion by the restriction endonuclease *Hae*III. 

Different patterns of DNA fragments result from SNP in the recognition site of *Hae*III. To design and use haplotype markers, the DNA fragments from the two constituent nuclei of the heterokaryotic strains, which were mixed in the PCR product, had to be sequenced after cloning. PCR products were cloned in *E. coli* XL1-blue (recA1 endA1 gyrA96 thi-1 hsdR17 supE44 relA1 lac [F′proAB lacIqZΔM15 Tn10 (Tetr)]) [27], using the plasmid vector pGEM-T-easy (Promega, Madison, WI, USA), according to the supplier’s recommendation. Chemo-competent bacteria were obtained as described by Hanahan [28]. The different sequences of ITS or *fruk* gene are haplotypes characterized by their nucleotides at all the SNPs found in these sequences.

### 2.5. Mitochondrial Marker

The design of the mitochondrial marker is based on previous data of Jalalzadeh et al. [25] for the three wild strains. After PCR amplification of *iAbi11* intron of the *cox1* gene and electrophoresis as indicated above, the lengths of amplified fragments for strains Bs243, Bs177, and Bs256 were 0 (lacking intron), 987 (intron carrying a potentially functional *heg*), and 2230 nt, respectively. In the latter case, the resident *heg* of the *iAbi11* intron would itself be invaded by a mobile ORF.

## 3. Results

### 3.1. Characterization of the Wild Heterokaryons Bs177, Bs243 and Bs256

#### 3.1.1. Different Cap Colors Observed in the Three Parental Strains

**Figure 2:**
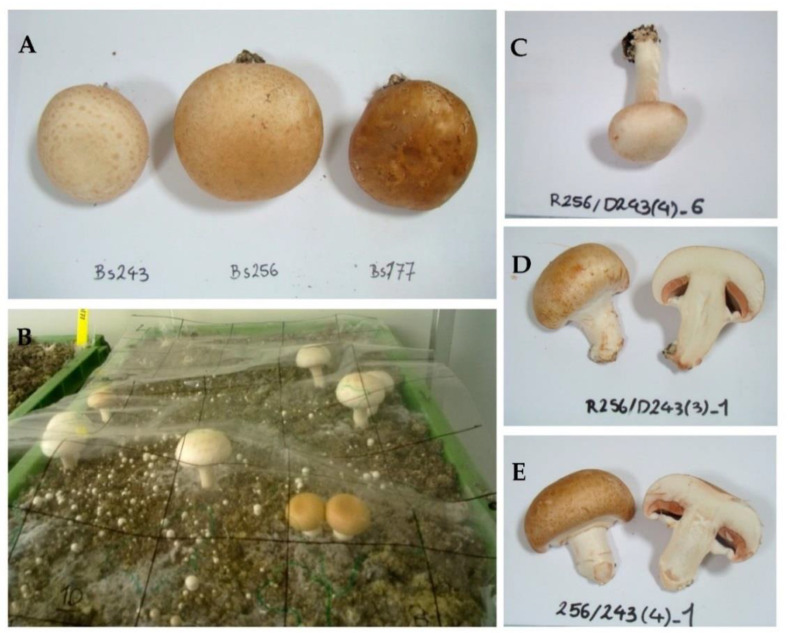
Basidiomata of parental strains (**A**) or growing on a culture tray of Bs256 having received propagules from Bs243 (**B**–**E**). (**A**) Basidiomata of Bs243, Bs256, and Bs177 have cream, medium brown and brown caps, respectively; (**B**) basidiomata of different phenotypes and development stages growing on a culture tray—in order to analyze a representative sample spread over the entire tray, basidiomata were collected as much as possible from the different plots drawn on the transparent plastic sheet; (**C**) basidioma (4)6 with cream-colored cap, such as Bs243, and the hybrid genotype *its*-5 *fruk*-4 + *its*-4 *fruk*-3; (**D**) basidioma (3)1 with medium brown cap, such as Bs256, and the hybrid genotype *its*-5 *fruk*-4 + *its*-3 *fruk*-2.; (**E**) basidioma (4)1 with medium brown cap, such as Bs256, and the same genotype as Bs256 (*its*-5 *fruk*-4 + *its*-5 *fruk*-5). All basidiomata collected on the trays of Bs256 had genotypes and phenotypes similar to those in photos C, D, or E. (see also Table 4).

Basidiomata of the three strains Bs177, Bs256 and Bs243 used in this study exhibited different cap colors in cultivation. They are dark brown, medium brown, and cream, respectively (Figure 2A).

#### 3.1.2. Detection of 29 SNPs and 24 Heteromorphisms in the ITS DNA Region and the *Fruk* Gene

The sequenced ITS and *fruk* DNA segments were 721 and 840 bp in length, respectively. As reported in a previous study [25] a high level of polymorphism was observed in the three strains Bs177, Bs243 and Bs256. Comparative sequence alignment of ITS region and *fruk* gene revealed 7 and 22 single nucleotide polymorphism (SNP) positions, respectively.

SNPs were numbered according to their position in the 5′3′sequenced fragments of ITS and *fruk* and detailed in Table 1 and Table 2, respectively. In addition, Bs177, Bs243 and Bs256 were heterozygous at 5, 3, and 0 of their 7 SNP positions of ITS sequence, respectively (Table 1) and at 0, 8, and 8 of their 22 SNP positions of *fruk* sequence, respectively (Table 2). These heteromorphisms appeared as double peaks in chromatograms of both strand sequences. The interpretation of doubles peaks as the presence of two nucleotide alleles at the same position was confirmed in the sequences from the PCR clones.

#### 3.1.3. Development of CAPS Nuclear Markers and Genotypes of the Parent Strains

CAPS makers were based on two SNPs (C/T). One is at positions 541 of the ITS sequence (locus *its*:541) and the other is at position 655 of the *fruk* sequence (locus *fruk*:655). As explained in detail in Table 1 and Table 2, recognition site (GGCC) for the endonuclease *Hae*III is only available when nucleotide C is present at these loci. Therefore, after digestion by *Hae*III and electrophoresis different patterns of DNA fragments can be observed. These patterns and the genotypes are reported in detail in Table 1 and Table 2. Genotypes of Bs177, Bs243, and Bs256 at loci *its*:541 and *fruk*:655 are *its*:541-2/2 *fruk*:655-1/1, *its*:541-1/2 *fruk*:655-1/2, and *its*:541-1/1 *fruk*:655-1/2, respectively. Once developed for parental strains, CAPS markers can be used to quickly screen genotypes among a large number of samples, offspring or hybrids by simply digesting PCR products and running electrophoreses.

#### 3.1.4. Development of Haplotype Nuclear Markers and Haplotypes Found in the Parent Strains

Haplotype nuclear markers were based on the haploid DNA sequences obtained after PCR cloning of ITS, which varied at 7 SNPs, or those of *fruk* gene, which varied at 22 SNPs. For each marker and each parental strain, inserts carried by four recombinant plasmids were sequenced. They were used to characterize the two component nuclei of each of the three wild strains (Table 1 and Table 2). Through PCR cloning, five haplotype profiles of ITS, numbered *its*-1 to *its*-5, were found in the six constituent nuclei of the three wild strains and are reported in Table 1. For the *fruk* gene, five haplotype profiles (*fruk*-1 to *fruk*-5) were found and reported in Table 2. These ITS and *fruk* haplotype profiles are useful markers for our purpose because, following a single meiotic generation, they should remain intact in the descendants of the three strains or the hybrids occurring between them. Indeed, crossovers between SNPs in ITS or *fruk* sequenced DNA segments is unlikely not only because of the short genetic distance between them but also because intrachromosomal recombination (crossovers) is suppressed in *A. bisporus* var. *bisporus* [29]. However, during meiosis, inter-chromosomal recombination should randomly occur between ITS and *fruk* markers which are found in Chromosome IX [30] and are *MAT*-linked on Chromosome I [24], respectively. Genotypes of Bs177, Bs243, and Bs256 at *its* and *fruk* haplotype markers are *its*-1/2 *fruk*-1/1, *its*-3/4 *fruk*-2/3, and *its*-5/5 *fruk*-4/5, respectively.

Sequences of *its* and *fruk* gene of the three wild strains have been previously deposited in GenBank [25] are recalled in Table 1 and Table 2. Sequences of 10 haploid clones with the five haplotype profiles of the ITS region and the five haplotype profiles of the *fruk* gene have been deposited in GenBank and their accession number are also in Table 1 and Table 2.

#### 3.1.5. Haplotype Mitochondrial Marker with Different Lengths in the Three Parental Strains

The mitochondrial marker is based on the length of the PCR products of the *iAbi11* intron of *cox1* gene as previously reported by Jalalzadeh et al. [25] and in the Materials and Methods section above. Here, we confirmed these data and assigned the haplotype profiles *iAbi11-S* (S = small intron), *iAbi11-0* (0 = no intron), and *iAbi11-L* (L = large intron), to strains Bs177, Bs243, and Bs256, respectively.

### 3.2. Characterization of Basidiomata Collected on the Receiver Bs177

In total, 50 basidiomata (25 per tray) were collected for the receiving strain Bs177. Basidiomata were harvested every day for one month and as much as possible over the entire compost surface. All the basidiomata were dark brown, as was the receiving strain Bs177.

In this experiment the donor was Bs243 and 26 of the 50 basidiomata collected on the trays of the receiver Bs177 were analyzed. For the CAPS markers, all had the same nuclear genotype as the receiver Bs177 *its*:541-2/2 *fruk*:655-1/1, the same mitochondrial haplotype *iAbi11*-S, and the same dark brown color of the cap. Despite the fact that we did not observe any hybridization events, after PCR amplification we sequenced 6 of these 26 samples and confirmed that their genotypes at the two haplotype markers were *its*:541-1/2 and *fruk*:655-1/1 as in Bs177. We conclude that hybridization did not spontaneously occur in this experiment. 

### 3.3. Characterization of Basidiomata Collected on the Receiver Bs256

#### 3.3.1. Cap Color, Mitochondrial Haplotypes and CAPS Nuclear Genotypes of 32 Basidiomata Collected on Bs256

In this experiment the donor was also Bs243. A total of 50 basidiomata (25 per tray) were collected for receiving strain Bs256. Trays were divided in small plots as shown in Figure 2B. Basidiomata were harvested, every day during one month and, as much as possible, from each of the small plots in order to have a representative sampling distributed on the entire compost surfaces. Observing some variations among the cap colors of the fruiting bodies growing on the receiver trays, a sample of 32 basidiomata selected at random among the 50 collected basidiomata was first analyzed with the mitochondrial marker and the nuclear CAPS markers (Table 3). Their cap colors are also indicated in this table: 14 had a medium brown cap and 18 had a cream cap.

All 32 basidiomata inherited the mitochondrial haplotype *iAbi11-L* of the receiver Bs256. The CAPS markers revealed two genotypic classes. One class includes 18 basidiomata with the genotypes *its*:541-1/2 *fruk*:655-2/2 and a cream cap color. The two constituent nuclei of these fruiting bodies had the genotypes *its*:541-1 *fruk*:655-2 and *its*:541-2 *fruk*:655-2, respectively. Table 3 shows all the possible nuclei and thus is useful to reconstitute different possible scenarios. The nucleus *its*:541-2 *fruk*:655-2 came from spores or mycelium of the donor Bs243 because allele ITS:541-2 is not present in Bs256. In contrast, nucleus *its*:541-1 *fruk*:655-2, which could theoretically come from both parents likely came from Bs256 because *fruk* is tightly linked to *MAT* and these heterokaryons should not have received two times the same *fruk* allele and thus the same mating type allele from the same parent Bs243. In conclusion, these 18 basidiomata are hybrids bearing the mitochondria and one nucleus of the receiver, whereas the second nucleus came from the mycelium or the spores of the donor. In the other genotypic class, the 14 remaining basidiomata had the genotype *its*:541-1/1 *fruk*:655-1/2 with the nuclear constituent genotypes *its*:541-1 *fruk*:655-2 and *its*:541-1 *fruk*:655-1. Despite they had a cap color similar to the receiver and also its mitochondrial haplotype *iAbi11-L*, Table 3 shows that the genotypes of their two constituent nuclei could originate from the receiver Bs256 as well as the donor Bs243. Therefore, using only these CAPS markers it was not possible to know if the basidiomata of this genotypic class were hybrid or not.

#### 3.3.2. Haplotypes of Basidiomata Collected on Bs256 Reveals Many Hybrids but with Only Two Genotypes in Agreement with the Involvement of Airborne Mycelium Fragments

Twelve basidiomata, six of each two genotypic classes described above using the CAPS markers, were analyzed with haplotype nuclear markers using sequencing after PCR amplification. The six samples which had hybrid genotypes with CAPS markers and a cream cap all had the same haplotypes *its*-4/5 *fruk*-3/4. In Table 4 this genotype is easily interpreted as a hybrid with a nucleus bearing Bs243 donor-specific haplotypes *its*4 *fruk*3 and another nucleus bearing Bs256 receiver-specific haplotypes *its*5 *fruk*4. One of these hybrid basidiomata is shown in Figure 2C. Among the six other samples exhibiting a medium brown cap color and that we could not identify as receiver or as hybrid using the CAPS markers, we found two different genotypes with the haplotype markers. Four were heterokaryons of hybrid type with genotype *its*-3/5 *fruk*-2/4. One of these hybrid basidiomata is shown in Figure 2D. As for the first group of hybrids, this second group of hybrids is interpreted in Table 4 as the association of a nuclei bearing Bs243 donor-specific haplotypes *its*3 *fruk*2 and nuclei bearing Bs256 receiver-specific haplotypes *its*-5 *fruk*-4. We note that the latter nucleus was present in all hybrids of both groups. The two remaining medium brown samples had the genotype of the receiver *its*-5/5 *fruk*-4/5 (nuclei *its*-5 *fruk*-4 + *its*-5 *fruk*-5). Since both nuclear and mitochondrial haplotypes as well as the cap color were consistent with the receiver, these two basidiomata were likely fruiting bodies of the receiver Bs256. One of them is shown in Figure 2E. In conclusion, two of the twelve basidiomata analyzed using haplotype nuclear markers were simple fruiting bodies of the receiver Bs256. The ten other samples were hybrids having both the same mitochondrial haplotype *iAbi11-L* and the same nuclear haplotype *its*-5 *fruk*-4 of the receiver Bs256. However, 6 of the 10 hybrids received the haplotypes *its*-4 *fruk*-3 from Bs243, whereas the remaining 4 hybrids received the haplotypes *its*-3 *fruk*-2 from Bs243. As indicated in Table 4, *its*-4 *fruk*-3-and *its*-3 *fruk*-2 could simply be the genotypes of the two constituent nuclei of the donor Bs243. In this scenario hybridizations occurred between pieces of mycelium from Bs243 and the mycelium of the receiver Bs256.

ITS and *fruk* sequences of two hybrid basidiomata H10 and H17 belonging to the two genotypic classes with the haplotypes *its*4/5 *fruk*3/4 and *its*3/5 *fruk* 2/4, respectively, were deposited in GenBank. The accession numbers are in Table 4.

#### 3.3.3. Different Results Confirming the Non-Involvement of the Spores in the Outcrossing

Using haplotype markers, we concluded above that hybrid basidiomata collected on the Bs256 trays likely resulted from crosses between Bs256 and airborne pieces of mycelia from Bs243. In an alternative interpretation, hybridizations would have occurred between Bs256 mycelium and spores or mycelia from spores of Bs243. However, this latter scenario is unlikely for the four following reasons.

First, knowing that the *fruk* gene and the *MAT* locus are tightly linked, the fact that all hybrids possessed the *fruk*-4 nuclear haplotype of the Bs256 receiver and received either *fruk*-2 or *fruk*-3 from the Bs243 donor implies that *fruk*-2, *fruk*-3, and *fruk*-4 are linked to three different mating type alleles. Since there is no sexual incompatibility, if the crosses had occurred via basidiospores, we should have observed four genotypic classes of hybrids that would have received nuclei with genotypes *its*-3 *fruk*-2 or *its*-4 *fruk*-3 (presumably parental genotypes) as well as genotypes *its*-3 *fruk*-3, or *its*-4 *fruk*-2 (presumably recombinant types) from donor Bs243 (Table 4). We not only observed only two classes of hybrids, but these two classes had received nuclei from Bs243 with the *its*-3 *fruk*-2 or *its*-4 *fruk*-3 genotypes that are complementary and thus likely those of the two constituent nuclei of the mycelium of Bs243.

Second, the same reasoning retrospectively applies to the larger sample of 32 basidiomata collected on Bs256 trays and analyzed with the CAPS markers. Basidiospores of the donor Bs243 should contains nuclei with four possible different genotypes as shown in Table 3, theoretically in equal proportions after meiosis. Among the 32 basidiomata, we found 22 hybrids belonging to two genotypic classes (18 with one genotype detected using CAPS markers and four genotypes detected later using haplotype markers). This absence of two classes of recombinant genotypes is in agreement with involvement of pieces of donor mycelium in the crosses, whereas four classes would be expected if the crosses had occurred with spores.

Third, hybrids receiving *its*-4 *fruk*-3 or *its*-3 *fruk*-2 nuclei from Bs243 had a cream or medium brown cap, respectively (Figure 2C,D). Such co-segregation for this trait with ITS region and *fruk* gene should not be expected if these nuclei came via basidiospores of Bs243. Indeed, there are several QTL implicated in the expression of the cap color trait and a major locus PPC1 that is on Chromosome VIII and thus non-linked to ITS or *fruk* [31,32]. In contrast, if the hybrids resulted from crosses between parental mycelia Bs243 and Bs256, those having both the same *its* and *fruk* haplotypes should also have the same nuclei and thus the same cap color genotype and phenotype. In fact, all hybrids of each of the two types have theoretically identical genomes except if mutation or somatic recombination occurred [17].

Fourth, the involvement of the pieces of mycelium is perfectly plausible since a lot of both spores and mycelium fragments were equally distributed on the trapping adhesive strips that were placed along the receiving trays. Roughly, about 50 of each were observed in the same microscopic field along all the adhesive strips and therefore the entire length of two receiver culture trays (2 × 60 = 120 cm in length) in each of two plastic tunnels. The length of the pieces of mycelium varied from 7µm, which is also the mean length of the spores, up to 45 µm.

In conclusion, under the conditions of our experiment, among the airborne propagules dispersed on the receiver trays, mycelium fragments were surprisingly as numerous as spores. We tested outcrossing for two pairs of donor and receiver strains. For one pair, all basidiomata growing on the receiver trays were fruiting bodies of the receiver. For the other pair, most basidiomata were hybrids with only two types of genotypes likely resulting from outcrossing involving airborne mycelium fragments. All the analyzed hybrids had the mitochondrion and one nucleus (always the same) from the receiver, whereas its other nucleus was one of the two presumed constituent nuclei of the donor. All data can be simply explained by crossing with mycelium fragments without any evidence of spore involvement.

However, half of the spores could have potentially produced hybrids with similar genotypes as those produced by mycelium fragments. Therefore, we cannot exclude that some of the evidenced hybrids resulted from outcrossing between spores of the donor and mycelium of receiver.

## 4. Discussion

### 4.1. Conditions for Outcrossing and Consequences on Genetic Diversity in Collection Sites

Our general goal was to understand how outcrossing naturally does occur in *A. bisporus* var. *bisporus*, knowing that the preferred life cycle of this variety is mainly pseudohomothallic and thus theoretically does not favor outcrossing. To do so, we simulated a situation in which crosses were promoted between airborne propagules from a fruiting donor mycelium and a receiver mycelium that invaded the culture substrate but had not yet fruited. Indeed, when the temperature and humidity remain relatively high after substrate invasion, there is a phase during which an aerial mycelium covers its surface. This aerial vegetative phase that is known in culture but that we have also observed in the wild has no nutritional interest for a secondary decomposer species such as *A. bisporus*. Thus, we suspected that it could be a phase naturally favoring the emission of mycelium fragments or the reception of propagules and consequently outcrossing. It was the challenge of our experiment to use this vegetative phase and the time lag between the fruiting of two strains to promote spontaneous hybridization between them. At the time of this phase, a cloud of propagules consisting of spores and mycelium fragments of the donor mycelium was aerially disseminated onto the receiver. The conditions for mycelium growth, fruiting, propagule dissemination and isolation were appropriate since we successfully collected hybrid fruiting bodies and they did not possess any unexpected alleles not belonging to the parental strains. This indicates that our experiment was somewhat secure, without crosses with unexpected propagules and without interaction between the two couples of parents which were isolated in two plastic tunnels. Similar and simultaneous processes were applied for both donor/receiver pairs Bs243/Bs177 and Bs243/Bs256 but hybrids were detected only among basidiomata collected on the receiver Bs256 and they only had two different genotypes. These experiments prove that outcrossing spontaneously occurs between strains growing in close proximity to each other. Given the predominantly pseudohomothallic life cycle of *A. bisporus* var. *bisporus*, outcrossing could be considered infrequent, but studies of local populations have shown a high level of diversity and heterozygosity with infrequent clonal or pseudoclonal lineages, suggesting that outcrossing may not be so rare [12]. In this latter study strains from specimens collected at less than one meter from each other were often genetically different suggesting that, except in rare cases, mycelium of *A. bisporus* generally extend to a small area in nature. However, in light of the present study strains isolated from specimens collected in the wild could have themselves resulted from hybridization between strains of the same collection site and thus the area of the resident mycelium could be larger than estimated from the genotypes of collected specimens. In addition, different genotypes and even different varieties or even species of Agaricus are frequently found close to each other probably because a resident mycelium can stimulate spore germination [33] even between species [34]. In addition, the fruiting of the strains in a same site is not necessarily synchronous since for example their ability to fruit at different temperatures is a variable trait [35]. These comments suggest that our outcrossing experiment conditions were realistic and helpful to explain the high diversity and heterozygosity found among wild basidiomata collected in previously studied natural collection sites.

### 4.2. Genotypes and Cap Color of the Hybrids

Genotype analysis using a mitochondrial marker based on intron presence/absence and two nuclear haplotypes markers based on multiple SNPs revealed that most basidiomata collected on the receiver trays invaded by Bs256 were hybrids that all inherited both its mitochondrial haplotype (*iAbi11-L*) and one of its two constituent nuclei that possesses the *its*-5 and *fruk*-4 haplotypes. The remaining constituent nucleus of the hybrids was one of the two constituent nuclei of the donor Bs243 that likely possess either the haplotypes *its*-4 and *fruk*-3 or *its*-3 and *fruk*-2. In addition, hybrids with the former haplotypes exhibited a cream cap like the donor parent, whereas those receiving the latter haplotypes exhibited a medium brown cap like the receiver parent Bs256. Therefore, the cap color of the later hybrids was not distinguishable from those of the basidiomata of their parent Bs256 fructifying on the same culture tray at the same time. Although data remained insufficient to hypothesize the genetics of cap color, this trait did identify one of the two classes of hybrids. The two hybrid genotypes and the absence of recombinants completely agree with the hypothesis that hybrid basidiomata resulted from crosses between the receiver BS 256 and only mycelium fragments of the donor Bs243 and not its spores.

### 4.3. How Did Outcrossing Occur?

The question arises as to why no hybrid or only hybrids with two genotypes were found among the basidiomata collected on the receiver trays. In first, the absence of hybrid basidiomata in the case of the couple Bs243/Bs177 suggests either a total incompatibility between these two strains and that outcrossing never occurred or hybridization occurred but the hybrid was not able to fruit when the receiver Bs177 was installed in the substrate. In this case, a problem of incompatibility might exist between the new hybrids and the resident mycelium. Genetics of vegetative incompatibility remain unknown in *A. bisporus*. In the case of the couple Bs243/Bs256, most of the fruiting bodies were hybrid basidiomata; however, they only had two genotypes, and we concluded that crosses had occurred between the mycelium of the receiver and pieces of mycelium of the donor, which were abundant among the airborne propagules with variable lengths (7–45 µm). Outcrossing was evidenced by analyzing the basidiomata. How this occurred remains unknown but we can propose some hypotheses. The fact that both mycelia of the donor and the receiver were involved simply suggests that crosses may have occurred between heterokaryons. It is the first time that outcrossing between heterokaryons is reported in situ. Crosses between heterokaryons of *A. bisporus* were first evidenced by Raper et al. [9] who observed that pairings of heterokaryons that were homoallelic for complementary auxotrophic mutations produced stable, prototrophic heterokaryons when nutritional selection was applied. 

It could be hypothesized that deheterokaryotisation would have occurred through mycelium fragmentation and that, in this case, crosses might occur between homokaryotic and heterokaryotic mycelia following a process called the Buller phenomenon [16]. We note that for deheterokaryotisation (=homokaryotisation) Dickhardt [36] fragmented a heterokaryotic mycelium with a razor blade and treated the mycelium with solutions of peptone or glycine. Conversely, Buller phenomenon could occur between heterokaryotic fragments of the donor mycelium and haploid hyphal tips of the receiver heterokaryon in which one of the two constituent nuclei would be more frequent than the other, which could bear a lethal or deleterious recessive allele. In this scenario, one of the two nuclei of the donor would simply migrate in the hyphal tip of the receiver. In addition, this would explain why always the same nucleus of the receiver is found in all hybrids, whereas the two nuclei of the donor were found. Another explanation should be some incompatibility between one nucleus of Bs256 and those of the donor. Moreover, it is possible that the two constituent nuclei of the receiver were involved in outcrossing; however, hybrids carrying one of the two nuclei grew or fruited much better than those carrying the other.

Another question is when does outcrossing occur? If mycelium fragments are involved, it should not necessarily occur during the fruiting process as in our experiment, but at any time when the aerial mycelium rises to the soil surface in warm, humid weather as indicated above in the conditions for outcrossing. In temperate north hemisphere, the period following the summer thunderstorms (August/September; too hot for fruiting) and proceeding the fruiting season (mainly November for *A. bisporus*) may be favorable. Therefore, it could exist different seasons favorable for outcrossing or for fruiting.

### 4.4. Success of Airborne Mycelium Fragments

In our experiment, airborne mycelium fragments unexpectedly played a major role, whereas we cannot exclude that some hybrids resulted from outcrossing involving spores. Conidia have never been observed in *A. bisporus* but pieces of mycelium can easily play this role. In addition, from our experience and that of mushroom growers, we know that mycelium of *A. bisporus* resists for several years in dry conditions. Nothing is known about these pieces of mycelium. Their quantity and role have been highly under-estimated until now. Such propagules could be involved not only in outcrossing but also in horizontal transfer through anastomosis for example of virus X in *A. bisporus* [37] or mitochondria in *A. subfloccosus* [38]. They will have to be examined in more detail in the future to better understand their number of cells and of nuclei, as well as their perennation and origin. They may have detached from the surface of the casing layer or from the fruiting bodies, mainly from their basis or their veil. We have not taken any specific action to produce them or increase their number.

One explanation of the success of the pieces of mycelium is maybe the timing of our experiment. Among propagules from Bs243 disseminated on Bs256, spores were also abundant and, thus, there was possibly a factor favoring the pieces of mycelium. Indeed, in the fruiting room, when the donor trays were enclosed in the plastic tunnel with the receiver trays, the latter, which was already completely invaded by the receiver mycelium, immediately received both types of propagules (pieces of mycelium and spores), and this for seven days (Figure 1A,B). Pieces of mycelia could be favored because they were immediately operational while spores needed to germinate, which take at least one week. In previous experiments of co-cultivation of spores and homokaryotic mycelium, Buller phenomenon occurred very quickly [19] with the heterokaryotic spores or mycelia from them but this does not represent a counter-example because mycelium fragments were not introduced and there was no competition between these two types of propagules. However, it should be noted that this previous study and the present study suggest that heterokaryons are involved in outcrossing and that this may be common in *A. bisporus*. Through such outcrossing with heterokaryons, haploid selection, commonly called gametic selection is bypassed and, as a result, deleterious or lethal recessive alleles could be accumulated. Furthermore, in *A. bisporus*, most of the spores are heterokaryotic like the mycelium fragments and thus these two predominant types of propagules contribute to the maintaining of these alleles.

In the future, the respective role of spore and mycelium fragments should be evaluated. Based on the new information provided by this first approach to understanding outcrossing processes, it may make sense to use parents with better known constituent nuclei for their mating types, as well as additional independent markers to analyze outcrossing events. Vegetative incompatibility between parental heterokaryons should be assessed in vitro.

### 4.5. Possible Undetected Recombination and Hidden Outcrossing

We did not observe recombination between the two markers located on different chromosomes in agreement with the fact that meiotic spores were not involved in the crosses. However, this is not a strict rule since Xu et al. [17] evidenced somatic recombination in confrontation between heterokaryons or between homokaryon and heterokaryon. Maybe by using larger sets of markers located on all chromosomes, some somatic recombinants could be found and thus more than two genotypic classes of hybrids. However, we have not currently detected such parasexuality. Furthermore, by using only four clones for each PCR product, we were fortunate to find both haplotypes of each parental strain without amplification artifacts that might be relatively frequent with such highly polymorphic sequences [39]. We must stay modest in our conclusions because reproduction remains hidden in cryptogams. For example, we detected only two types of hybrids mycelia maybe thanks to their rapid fruiting. We cannot exclude that other types of crosses involving the other nucleus of the receiver or spores from the donor could have produced other types of hybrids. Such hybrids could fruit later through various scenarios as for example when the resident mycelium will die or will be replaced, in a following season or in another place after a further aerial dissemination without any fruiting process.

### 4.6. A method for Strain Hybridization

Our objective was to experiment outcrossing as it presumably occurs in the wild. If the objective would have simply been to obtain hybrid basidiomata, our data suggest that a simple inoculation of the donor mycelium in the casing layer of the receiver trays could produce same hybrids as in our experiment. This could be a simpler but less efficient method than the one proposed by Callac et al. [19] who obtained various hybrids, after co-inoculation of the substrate simultaneously with spores and a homokaryotic mycelium. The latter method, which can be used for breeding [40], is efficient but more difficult to implement because it requires a homokaryon and drastic conditions to avoid uncontrolled hybridization of the homokaryon cultivated on compost substrate by unwanted inoculum. In any case, these outcrossing methods are not completely performed under axenic conditions and require more precautions than standard cultivation of the button mushroom.

### 4.7. Biotrophic Contribution of the Resident Mycelium

In *A. bisporus*, nuclear migration rarely occurs through the septa of either homokaryotic or heterokaryotic hyphae, which are both multinucleate and without clamp connection [9,41,42]. In other terms, whatever the hybridization process, the resulting heterokaryon is a new mycelium. In contrast, in many species exhibiting clamp connections heterokaryotisation of homokaryotic mycelium occurs through nuclear migration. However, although nuclei rarely migrate, anastomoses between the new hybrid heterokaryon and its parental homokaryon or heterokaryon are useful to feed the new hybrid and, therefore, increase its growth rate and fruiting. This is even necessary when the parental mycelium has already invaded all the substrate or in any asymmetric situation as in our experiment in which propagules from one parent are disseminated on another parent. The latter is the resident parent which is already installed and called the receiver in our experiment. From an ecological point of view the hybrid heterokaryon has biotrophic mycoparasitic relationships [43] with its parental resident mycelium. Different cases of trophic contribution of the resident mycelium to the fruiting of another newly introduced mycelium are reported in literature and listed in Savoie et al. [3] such as for example in experimental transplantation of basidiomata [44], or in cultivation of non-transgenic mycelium on a transgenic mycelium with production of non-transgenic basidiomata exhibiting the transgenic phenotype [45]. The biotrophic contribution of the resident mycelium would have two main consequences: (i) the vegetative incompatibility issues would not only concern the compatibility between the parents or the constituent nuclei of the hybrid but also the compatibility between the new hybrid and its parental resident mycelium; (ii) the trophic contribution from the resident mycelium to the hybrid mycelium is likely facilitated or even only possible if both have the same mitochondria.

Our experiment can be compared to a previous study in which a parental homokaryotic strain was co-inoculated as grain spawn in the culture substrate simultaneously with spores (mainly heterokaryotic) from another parental heterokaryotic strain. All the fruiting hybrids possessed the nucleus and the mitochondria of the inoculated homokaryotic strain and resulted from crosses with heterokaryotic spores or mycelium from the latter [19,40]. Although experimental conditions where different and the inoculated mycelium invading early the culture substrate was homokaryotic in the previous study and heterokaryotic in the present experiment, it acted as resident mycelium in both situations. Indeed, in both experiments, each of the hybrid basidiomata had one nucleus and mitochondria from their parental resident mycelium and another nucleus from propagules of their other parent, which were heterokaryotic spores and pieces of mycelium, respectively. In each of these situations, different modes of outcrossing are likely to occur, but as long as the resident mycelium is ubiquitous in the substrate or soil, the earliest hybrids possessing the same mitochondria as it would have an advantage in growing and fruiting. In the future, the biotrophic role of the resident mycelium and the preferential inheritance of its mitochondria in the hybrids will need to be confirmed under other outcrossing conditions or simply by testing other pairs of parent mycelium. In our experiments, applying the same conditions and using two different pairs of strains, we observed two different results. In the wild, the potential relationships between a resident mycelium and the airborne propagules it receives are certainly very variable and probably many scenarios are possible.

### 4.8. Conclusions

When we designed the experiments, we expected to collect basidiomata on the receiver trays which were produced either by the receiver mycelium or by hybrid mycelia between the receiver and propagules from the donor. We tested two pairs of parents in the same conditions. In both cases, about half of the propagules were mycelium fragments and half were spores but in one case none of the basidiomata was hybrid, whereas in the other case, most basidiomata were hybrids. Among the hybrids only two genotypes were found. All these hybrids shared the mitochondia and one nucleus of the receiver and one of the two presumed constituent nuclei of the donor. The absence of recombinant nuclei from the donor revealed that airborne mycelium fragments rather than spores were involved in outcrossing. Therefore, outcrossing can easily occur in the wild between heterokaryotic mycelia growing in close proximity to each other. The success of the mycelium fragments is possibly due to their resistance to dryness and their activation probably faster than that of the spores when these propagules are disseminated on a resident mycelium. The comparison with previous experiments suggests that the inheritance of the mitochondria of the receiver resident strain should favor its biotrophic contribution to the growth of the new hybrid mycelium and its fruiting.

*A. bisporus* var. *bisporus* was considered as a pseudohomothallic species in which outcrossing should unfrequently occur. Our data show that outcrossing involving mycelium fragments could easily occur and thus contribute to the high level of heterozygosity and the cultivar introgression previously reported in wild populations. We do not claim to have elucidated all the allogamous processes that occur in *A. bisporus* var. *bisporus*, but we believe we have uncovered an effective and unexpected process that could impact population dynamics and structure.

## Figures and Tables

**Table 1:** Genotyping of three wild strains, either using ITS haplotypes or using the CAPS marker *its541.*

Polymorphic Positions	Genotyping Based on ITS Sequences Obtained after PCR Cloning ^1^								Genotyping at *its:*541 CAPS Marker after *Hae*III Digestion ^2^		
	ITS Haplotypes of the Nuclei Based on PCR Clones					Genotypes of the Strains			Genotypes of the Strains		
	*its-*1 Bs177-Clone1 OP518685	*its-*2 Bs177-Clone2 OP518686	*its-*3 Bs243-Clone1 OP518687	*its-*4 Bs243-Clone2 OP518688	*its-*5 Bs256 OP520895	*its-*1/2 Bs177 KF848690	*its-*3/4 Bs243 KF848693	*its-*5/5 Bs256 KF848696	*its:*541-2/2 Bs177	*its:*541-1/2 Bs243	*its:*541-1/1 Bs256
51	G	A	A	A	A	**A/G**	A/A	A/A			
169	T	C	C	T	C	**C/T**	**C/T**	C/C			
172	C	T	T	C	T	**C/T**	**C/T**	T/T			
503	G	A	G	G	G	**A/G**	G/G	G/G			
541	C	C	T	C	T	C/C	**C/T**	T/T	205, 244, 317 (C/C)	205, 244, 317, 522 (**C/T**)	244, 522 (T/T)
547	C	A	C	C	C	**A/C**	C/C	C/C			
582	C	C	C	C	T	C/C	C/C	T/T			

Heterokaryotic strains were genotyped from PCR products of ITS nuc rDNA, using the following two different approaches: ^1^ after sequencing of the PCR products and their clones, the heterokaryons and their nuclei were genotyped on the basis of nucleotides present at 7 SNP positions. Five haplotype alleles numbered *its-*1 to *its-*5 were identified. GenBank accession numbers of the parents and their clones are indicated; ^2^ after digestion of the PCR product with *Hae*III, electrophoresis revealed different patterns of DNA fragment. Presence of the C or T nucleotide at position 541 renders the site ggcC recognizable (allele *its:*541-2) or not (ggcT; allele *its*:541-1) by *Hae*III. Heteromorphisms are indicated in bold type.

**Table 2:** Genotyping of three wild strains, either using *fruk* haplotypes or using the CAPS marker *fruk655.*

Polymorphic Position	Genotyping Based on *fruk* Sequences Obtained after PCR Cloning ^1^								Genotyping at *fruk*:655 CAPS Marker after *Hae*III Digestion ^2^		
	*fruk* Haplotypes of Parental Nuclei					Genotype of the Strains			Genotypes of the Strains		
	*fruk*-1 Bs177 OP555287	*fruk*-2 Bs243-clone1 OP555288	*fruk*-3 Bs243-clone2 OP555289	*fruk*-4 Bs256-clone1 OP55529	*fruk*-5 Bs256-clone2 OP555291	*fruk*-1/1 Bs177 KF848701	*fruk*-2/3 Bs243 KF848704	*fruk*-4/5 Bs256 KF848707	*fruk*:655-1/1 Bs177	*fruk*:655-1/2 Bs243	*fruk*:655-1/2 Bs256
144	C	C	T	C	C	C/C	**C/T**	C/C			
242	C	G	C	G	G	C/C	**G/C**	G/G			
247	G	A	A	A	A	G/G	A/A	A/A			
279	C	T	T	T	T	C/C	T/T	T/T			
289	A	A	A	G	A	A/A	A/A	**A/G**			
435	C	C	T	T	C	C/C	**C/T**	**C/T**			
463	C	T	T	T	T	C/C	T/T	T/T			
490	C	T	T	T	T	C/C	T/T	T/T			
640	A	G	G	G	G	A/A	G/G	G/G			
655	T	T	C	C	T	T/T	**C/T**	**C/T**	879 (T/T)	205, 674, 879 (**C/T**)	205, 674, 879 (**C/T**)
671	C	T	T	T	T	C/C	T/T	T/T			
674	A	G	G	G	G	A/A	G/G	G/G			
676	C	C	A	C	C	C/C	**C/A**	C/C			
677	G	G	A	G	G	G/G	**A/G**	G/G			
683	G	A	G	G	A	G/G	**A/G**	**A/G**			
710	G	G	G	A	A	G/G	G/G	A/A			
730	C	G	G	C	C	C/C	G/G	C/C			
752	C	C	T	C	T	C/C	**C/T**	**C/T**			
788	G	A	A	G	A	G/G	A/A	**A/G**			
809	A	G	G	G	G	A/A	G/G	G/G			
815	G	G	G	A	G	G/G	G/G	**A/G**			
823	T	C	C	C	T	T/T	C/C	**C/T**			

Heterokaryotic strains were genotyped from PCR products of *fruk* gene, using the following two different approaches: ^1^ After sequencing of the PCR products and their clones, the heterokaryons and their nuclei were genotyped on the basis of the nucleotides present at 22 SNP positions. Five haplotypes (*fruk*-1 to *fruk*-5) were identified. GenBank accession numbers of the parents and their clones are indicated. ^2^ After digestion of the PCR products with *Hae*III, electrophoresis revealed different patterns of DNA fragments. Presence of the C or T nucleotide at position 655 renders the site ggCc recognizable (allele *fruk*:655-2) or not (ggTc; allele *fruk*:655-1) by *Hae*III. Heteromorphisms are indicated in bold type.

**Table 3:** Nuclear genotypes at CAPS markers and mitochondrial haplotypes in the experiment using Bs256 as receiver of airborne propagules from the culture trays of the donor Bs243.

Material	Ploidy Level	Expected and Observed *its* and *fruk* Genotypes at CAPS Markers in Mycelia and Spores	Mitochondrial Haplotype
Donor mycelium Bs243	Heterokaryotic mycelium	*its*:541-1 *fruk*:655-1 + ***its*:541-2 *fruk*:655-2** or *its*:541-1 *fruk*:655-2 + *its*:541-2 *fruk*:655-1	*iAbi11-0*
Basidiospores of Bs243 (possible genotypes)	Homokaryotic spores	*its*:541-1 *fruk*:655-1	*iAbi11-0*
		*its*:541-1 *fruk*:655-2	*iAbi11-0*
		*its*:541-2 *fruk*:655-1	*iAbi11-0*
		***its*:541-2 *fruk*:655-2**	*iAbi11-0*
	Heterokaryotic spores ^1^	*its*:541-1 *fruk*:655-1 + ***its*:541-2 *fruk*:655-2**	*iAbi11-0*
		*its*:541-1 *fruk*:655-2 + *its*:541-2 *fruk*:655-1	*iAbi11-0*
		*its*:541-1 *fruk*:655-1 + *its*:541-1 *fruk*:655-2 infrequent ^2^	*iAbi11-0*
		***its*:541-2 *fruk*:655-2** + *its*:541-2 *fruk*:655-1 infrequent ^2^	*iAbi11-0*
Receiver mycelium Bs256	Heterokaryotic mycelium	*its*:541-1 *fruk*:655-2 + *its*:541-1 *fruk*:655-1 (medium brown cap)	*iAbi11*-*L*
Basidiomata collected on Bs256 (32)	Heterokaryon of unknown type (14)	*its*:541-1 *fruk*:655-2 + *its*:541-1 *fruk*:655-1 (medium brown cap) ^3^	*iAbi11-L*
	Heterokaryon of hybrid type (18)	*its*:541-1 *fruk*:655-2 + ***its*:541-2 *fruk*:655-2** (cream cap)	*iAbi11-L*

^1^ Genotypes of spores homozygous at *fruk* are unlikely and not represented because non-sister meiotic nuclei are paired in the same spore and because this gene is linked to both the centromere and the mating type locus *MAT*. ^2^ Loss of parental heterozygozity at most loci, including *its*, is infrequent because non-sister meiotic nuclei are paired in the same spore and because recombination is suppressed in *A. bisporus* var. *bisporus.* ^3^ The nuclear genotypes could come from the donor as well as from the receiver. The nuclear genotype in bold type, which can come only from the donor, was found in hybrid basidiomata fruiting on the culture trays of the receiver Bs256.

**Table 4:** Nuclear and mitochondrial haplotypes of parental mycelia, expected spores, and collected basidiomata in the outcrossing experiment between Bs256 mycelium and airborne propagules from Bs243.

Material	Ploidy Level	Expected and Observed *its* and *fruk* Nuclear Haplotypes in Mycelia and Spores	Mitochondrial Haplotype
Donor mycelium Bs243	Heterokaryotic mycelium	***its*-3 *fruk*-2 + *its*-4 *fruk*-3** or *its*-3 *fruk*-3 + *its*-4 *fruk*-2	*iAbi11-0*
Basidiospores of Bs243 (possible genotypes)	Homokaryotic spores	***its*-3 *fruk*-2**	*iAbi11-0*
		***its*-4 *fruk*-3**	*iAbi11-0*
		*its*-3 *fruk*-3	*iAbi11-0*
		*its*-4 *fruk*-2	*iAbi11-0*
	Heterokaryotic spores ^1^	***its*-3 *fruk*-2 + *its*-4 *fruk*-**3	*iAbi11-0*
		*its*-4 *fruk*-2 + *its*-3 *fruk*-3	*iAbi11-0*
		(***its*-3 *fruk*-2** + *its*-3 *fruk*-3) infrequent ^2^	*iAbi11-0*
		(*its*-4 *fruk*-2 + ***its*-4 *fruk*-3**) infrequent ^2^	*iAbi11-0*
Receiver mycelium Bs256	Heterokaryotic mycelium	*its*-5 *fruk*-4 + *its*-5 *fruk*-5	*iAbi11-L*
Basidiomata collected on receiver trays (12)	Heterokaryon of parental type (2)	*its*-5 *fruk*-4 + *its*-5 *fruk*-5 (2 with medium brown cap)	*iAbi11-L*
	Heterokaryon of hybrid type (10)	*its*-5 *fruk*-4 + ***its*-3 *fruk*-2** (4 with medium brown cap) example: hybrid Bs256xBs243-H17 *its* 3/5 GB ^3^: OP704222, *fruk* 2/4 GB: OP750462	*iAbi11-L*
		*its*-5 *fruk*-4 **+ *its*-4 *fruk*-3** (6 with cream cap) example: hybrid Bs256xBs243-H10 *its* 4/5 GB ^3^: OP704221, *fruk* 3/4 GB: OP750461	*iAbi11-L*

^1^ Genotypes of spores homozygous at *fruk* are unlikely in *A. bisporus* and not represented because non-sister meiotic nuclei are paired in heterokaryotic spores and because this gene is linked to both the centromere and the mating type locus. ^2^ Loss of parental heterozygozity at most loci, including *its*, is infrequent because non-sister meiotic nuclei are paired in the same spore and because recombination is suppressed in *A. bisporus* var. *bisporus.* ^3^ GenBank (GB) accession numbers of ITS and *fruk* sequences of two basidiomata (H10 and H17) belonging to the two genotypic classes of hybrids are indicated The two genotypes in bold type are those of nuclei bearing donor-specific haplotypes and those that were found in the hybrid basidiomata collected on the culture trays of the receiver Bs256.

## Data Availability

The sequences newly generated in this study can be found in the NCBI database under the codes: OP518685, OP518686, OP518687, OP518688, OP520895, OP555287, OP555288, OP555289, OP555290, OP555291, OP704221, OP704222, OP750461, and OP750462.
